# Statin-Induced Increases in Atrophy Gene Expression Occur Independently of Changes in PGC1α Protein and Mitochondrial Content

**DOI:** 10.1371/journal.pone.0128398

**Published:** 2015-05-28

**Authors:** Craig A. Goodman, Derk Pol, Evelyn Zacharewicz, Robert S. Lee-Young, Rod J. Snow, Aaron P. Russell, Glenn K. McConell

**Affiliations:** 1 Department of Physiology, University of Melbourne, Parkville, Victoria, Australia; 2 Institute of Sport, Exercise and Active Living and the College of Health and Biomedicine, Victoria University, Victoria, Australia; 3 Centre for Physical Activity and Nutrition, School of Exercise and Nutrition Sciences, Deakin University, Burwood, Australia; 4 Cellular and Molecular Metabolism Laboratory, Division of Metabolism and Obesity, Baker IDI Heart and Diabetes Institute, Melbourne, Victoria, Australia; University of Louisville School of Medicine, UNITED STATES

## Abstract

One serious side effect of statin drugs is skeletal muscle myopathy. Although the mechanism(s) responsible for statin myopathy remains to be fully determined, an increase in muscle atrophy gene expression and changes in mitochondrial content and/or function have been proposed to play a role. In this study, we examined the relationship between statin-induced expression of muscle atrophy genes, regulators of mitochondrial biogenesis, and markers of mitochondrial content in slow- (ST) and fast-twitch (FT) rat skeletal muscles. Male Sprague Dawley rats were treated with simvastatin (60 or 80 mg·kg^-1^·day^-1^) or vehicle control via oral gavage for 14 days. In the absence of overt muscle damage, simvastatin treatment induced an increase in atrogin-1, MuRF1 and myostatin mRNA expression; however, these were not associated with changes in peroxisome proliferator gamma co-activator 1 alpha (PGC-1α) protein or markers of mitochondrial content. Simvastatin did, however, increase neuronal nitric oxide synthase (nNOS), endothelial NOS (eNOS) and AMPK α-subunit protein expression, and tended to increase total NOS activity, in FT but not ST muscles. Furthermore, simvastatin induced a decrease in β-hydroxyacyl CoA dehydrogenase (β-HAD) activity only in FT muscles. These findings suggest that the statin-induced activation of muscle atrophy genes occurs independent of changes in PGC-1α protein and mitochondrial content. Moreover, muscle-specific increases in NOS expression and possibly NO production, and decreases in fatty acid oxidation, could contribute to the previously reported development of overt statin-induced muscle damage in FT muscles.

## Introduction

Statin drugs lower blood cholesterol, and thus reduce the risk of coronary heart disease and stroke, by inhibiting the rate limiting enzyme of the mevalonate pathway, 3-hydroxy-3-methylglutaryl coenzyme A (HMG-CoA) reductase (for review see [1]). With reductions in low-density lipoprotein (LDL) cholesterol of up to 55%, statins have become the most commonly prescribed drug in the world today, with more and more populations being indicated for their use [2,3]. Although generally well tolerated, one of the main side effects of statin medications is skeletal muscle myopathy, with clinical symptoms that include muscle pain (myalgia), inflammation (myositis), weakness, fatigue and cramping [4,5]. With an incidence of >10% of statin users in the general population [6,7], hundreds of thousands of people worldwide are likely to experience some form of statin-induced myopathy. Statin related muscle symptoms also appear to be exacerbated by exercise [8]. Thus, statin-induced myopathy has the potential to markedly affect levels of physical activity and quality of life [9], and could prompt the discontinuation of the statin therapy altogether. Therefore, a thorough understanding of the molecular mechanism(s) underlying statin myopathy is essential for the future identification of specific biomarkers to detect adverse statin-induced events prior to the potential onset of debilitating clinical symptoms and/or for the development of safer alternative cholesterol lowering agents.

Although the exact mechanism(s) responsible for statin-induced myopathy remains to be definitively determined, recent studies have suggested that mitochondria and/or the activation of muscle atrophy-related genes may play a role (for reviews see [10–12]). For example, statins have been shown to up regulate the expression of the muscle-specific ubiquitin proteasome system (UPS) E3-ligases, atrogin-1 and MuRF1, in a range of model systems including statin-myopathy patients [13–18]. Importantly, statin-induced muscle atrophy/damage was markedly reduced in myotubes from atrogin-1 knockout mice and in Zebra fish transfected with atrogin-1 siRNA [14]. Together, these findings suggest that an increase in the expression of muscle specific E3-ligases (i.e. atrogin-1 and MuRF1) play a crucial role in statin-induced muscle fiber atrophy/damage and may help to explain muscle pain and weakness associated with statin-myopathy.

Numerous clinical, animal and cell culture studies have provided evidence that statin-myopathy is also associated with impaired mitochondrial function and morphology (e.g. [17–29]). In addition, recent studies also suggest that statins induce a reduction in mitochondrial content/volume [14,23,30–32]; an effect that could, in part, be due to reduced mitochondrial biogenesis. Mitochondrial biogenesis is positively regulated by a variety of signaling molecules and transcriptional co-activators, including the peroxisome proliferator gamma co-activator 1 alpha (PGC1α) [33]. For example, PGC1α binds to and co-activates nuclear respiratory factor 1 (NRF1) which, in turn, regulates the transcription of mitochondrial transcription Factor A (Tfam) [33]. Recent studies have reported a statin-induced reduction in PGC1α mRNA expression in humans, rodents and cultured cells [23,32]. Therefore, statins could down regulate mitochondrial biogenesis via a reduction in PGC1α expression. Importantly, PGC1α has also been proposed to play an inhibitory role against the activation of atrophy gene expression and muscle atrophy [14,34,35]. Thus, a statin-induced decrease in PGC1α protein and/or co-transcriptional activity could reduce mitochondrial biogenesis and also play a role in the induction of atrophy gene expression. To date, however, no studies have examined the relationship, if any, between changes in PGC1α protein, markers of mitochondrial content and the expression of atrophy genes in skeletal muscle with *in vivo* statin treatment.

In contrast to their effect on PGC1α expression, non-muscle studies have shown that statins also positively regulate two other important activators of mitochondrial biogenesis i.e. AMP-activated protein kinase (AMPK) [e.g. [36–39]], and nitric oxide (NO) via increases in endothelial (eNOS) and neuronal (nNOS) nitric oxide synthase isoform expression and activity [e.g.[40–43]]. Thus, statins may paradoxically also exert a stimulatory effect on mitochondrial biogenesis via an increase in AMPK and NOS activity. Interestingly, an increase in skeletal muscle AMPK activity can also activate the expression of atrogin-1 and MuRF1, and induce muscle fiber atrophy [for review see [44]], while nNOS has been implicated in the induction of atrogin-1 and MuRF1 gene in various muscle atrophy models [45,46]. To date, however, no studies have examined the effect of statins on AMPK or NOS expression in skeletal muscle *in vivo*.

Therefore, to gain a more complete understanding of the early molecular events associated with statin-induced myopathy, the purpose of this study was to determine whether statin-induced increases in muscle atrophy gene expression are associated with changes in: 1) PGC1α protein expression; 2) mitochondrial enzyme activity and mitochondrial protein expression; 3) AMPK protein expression and activation (phosphorylation); and 4) eNOS and nNOS protein expression and total NOS activity.

## Materials and Methods

### Animals

Male Sprague Dawley rats (6–7 wk; 214.8 ± 11.1g, mean ± SEM) were obtained from the Biological Research Facility, The University of Melbourne, Victoria, Australia and kept at 22 ± 2°C with *ad libitum* access to standard chow and water. All procedures used in this study were approved by The University of Melbourne Animals Experimentation Ethics Committee (Permit #- 0704504.5).

### Statin Dosage and Tissue Collection

In order to examine the early effect of statins on the induction of atrophy genes and signaling molecules involved in mitochondrial biogenesis, independent of overt muscle damage and regeneration, we employed doses of statin (simvastatin) that Mallinson et al. (2009) have previously shown to not induce overt damage and regeneration in rat skeletal muscle over a ~2 week period. Therefore, rats were divided into three groups of 8 and treated for 14 days with vehicle control or either 60 mg·kg^-1^·day^-1^ (Sim 60) or 80 mg·kg^-1^·day^-1^ (Sim 80) of simvastatin. (For a detailed rationale for this dosing range, see the Discussion section of Mallinson et al, 2009). Simvastatin (a gift from Pfizer Inc., Kent, UK) was suspended in 0.5% methyl cellulose and administered via oral gavage at a dose of 5.0 ml.kg^-1^. Simvastatin has a half-life of ~ 2 hr *in vivo* [47]. Vehicle control animals received 0.5% methyl cellulose vehicle by oral gavage at the same relative volume for 14 days. Food consumption was monitored daily and rat body mass was measured every morning prior to gavaging. On day 15, 24 hr following the last simvastatin or vehicle treatment, rats were killed by intraperitoneal injection of pentobarbital sodium (325mg.ml^-1^, Virbac, Australia) followed by cervical dislocation. Blood samples were immediately collected via cardiac puncture. Based on previous studies that have shown a greater susceptibility of fast-twitch muscles to statin-induced muscle damage/necrosis compared with slow-twitch muscles in rodents [25,48–50], we compared the effect of statin treatment between the fast-twitch extensor digitorum longus (EDL) and plantaris (PLT) muscles, and the slow-twitch soleus (SOL) muscle. As such, EDL, PLT and SOL muscles were rapidly collected and subjected to the various measurements described below.

### Blood Cholesterol and Creatine Kinase Analysis

Plasma [CK] and [total cholesterol] were measured by Melbourne Health Pathology Service (Royal Melbourne Hospital, Parkville, Victoria) using an Olympus 2700 Autoanalyser (Olympus Diagnostics, Clare, USA).

### Muscle Sample Preparation and Immunoblotting

For Western blot analysis, EDL, PLT and SOL muscles were crushed under liquid nitrogen and aliquots homogenized using a Polytron PT-MR 1200 (Luzernerstrasse, Switzerland) in 10 volumes of extraction buffer containing 50 mM Tris, 1 mM EDTA, 10% vol/vol glycerol, 1% vol/vol Triton X-100, 50 mM NaF, 5 mM Na_4_P_2_O_7_, 1 mM DTT, 1 mM PMSF, 10g/ml of Trypsin inhibitor (Sigma, St. Louis, MO, USA) and 5l/ml Protease Inhibitor Cocktail (P8340, Sigma, St. Louis, MO) (pH 7.5). The resulting lysates were left on ice for 20 min and then spun at 10,000 *g* for 20 min at 4°C. Protein concentration was determined using a bicinchoninic acid (BCA) protein assay (Pierce, Rockford, IL). Samples were then dissolved in Laemmli buffer and subjected to electrophoretic separation by SDS-PAGE. Following electrophoretic separation, proteins were transferred to a PVDF membrane, blocked with 5% powdered milk in PBST (Phosphate-buffered saline, 1% Tween 20) for 1 h followed by an overnight incubation at 4°C with primary antibody. Primary antibodies used were: rabbit anti-AMPK α-pan (1:1000, Cell Signaling), rabbit anti-Thr^172^ phospho-AMPK (1:1000, Cell Signaling), rabbit anti-COX4 (1:1000, Cell Signaling), mouse anti-CytC (1:1000, Cell Signaling), mouse anti-eNOS (1:1000, BD Bio Science), mouse anti-nNOS (1:1000, BD Bio Science), mouse anti-NRF-1 (1:1000, Rockland), mouse anti-PGC-1α (1:1000, Chemicon), and mouse anti-Tfam (1:1000, GenWay). The primary antibody was detected with IRDye^TM^ 800-conjugated anti-rabbit IgG (1:5000; Rockland, Gilbertsville, PA) or IRDye^TM^ 680-conjugated anti-mouse IgG (1:5000; Molecular Probes, Invitrogen) secondary antibodies and protein bands analyzed by infrared detection (Odyssey Imaging system, LI-COR Biosciences, Lincoln, NE). Membranes were reprobed with rabbit anti-GAPDH antibody (1:10000, Cell Signaling). To control for any differences in protein loading, the intensity of the protein band of interest was expressed relative to glyceraldehyde 3-phosphate dehydrogenase (GAPDH) band intensity from the same sample. Simvastatin had no effect on GAPDH protein abundance in any of the three muscles examined in this study (S1 Fig).

### RNA extraction, Reverse Transcription and qPCR

Approximately 8–12 mg of crushed muscle was homogenised using a FastPrep® instrument (Qbiogene, Seven Hills, NSW) followed by RNA extraction using TRIzol® reagent (Invitrogen, Mulgrave, VIC) [51] combined with the PureLink^TM^ RNA Mini Kit (Invitrogen, Mulgrave, VIC), including DNase treatment, as per the manufacturers instruction. RNA concentration and purity was determined using the NanoDrop 2000 spectrophotometer (NanoDrop products, Wilmington, DE). Samples were stored at -80°C until further use.

RNA was reverse transcribed to cDNA using Oligo-dT primers and the High Capacity RNA-to-cDNA kit (Invitrogen, Mulgrave, VIC). Following reverse transcription, the samples were treated with Ribonuclease H (RNase H) (Invitrogen, Mulgrave, VIC) at 37°C for 30 minutes to degrade any remaining RNA. Real-time polymerase chain reaction (RT-PCR) was performed using a Stratagene Mx3000 thermocycler and a Brilliant® Multiplex QPCR master mix (Integrated Sciences, Chatswood, NSW) as published previously [52]. The primer and probe sets for atrogin-1, MuRF-1 and myostatin are provided in Table 1. mRNA expression was measured in triplicate and normalised to total cDNA as determined using the Quant-it OliGreen ssDNA Assay Kit (Invitrogen, Mulgrave, VIC) [51].

**Table 1:** Primers and probes used in RT-PCR.

Target	Primer and probe sequence		Primer Concentration (nM)
Atrogin-1	**F**	5’ ATG CCG TTC CTT GGT CAG 3’	150
	**R**	5’ ACT GCT GAG GTC GCT CAC 3’	150
	**P**	5’ TGC CGC TTT TCT CAT CCA 3’	100
MuRF-1	**F**	5’ AGG ACT GAA TTT GTG TTA TAT GTT G 3’	150
	**R**	5’ TAG CCT CGA ACT CAT AGA GAT C 3’	150
	**P**	5’ AAC TGC CTC TGC CTC CA 3’	100
Myostatin	**F**	5’ AGA CAA CTT CTG CCC AGA G 3’	50
	**R**	5’ TCC GTG GTA GCG TGA TAA TC 3’	500
	**P**	5’ CCG TCA CTG CTG TCA TCC 3’	200

F, forward primer; R, reverse primer; P, probe

### Mitochondrial Enzyme Activity

Citrate synthase (CS) and β-Hydroxyacyl CoA dehydrogenase (β-HAD) activities in an aliquot of crushed muscle were measured spectrophotometrically as previously described [53]. Enzyme activities were measured using a Multiskan EX photometric microplate absorbance reader and Ascent software (Thermo Electron Corporation, Vantaa, Finland), and expressed per g of muscle (μmol.min^-1^.g^-1^).

### Nitric Oxide Synthase Activity

Total NOS activity was measured using 40–70 μg of protein as previously described [54]. NOS activity was measured in whole cell lysates as the difference in activity between samples incubated with or without *N* ^ω^-nitro-_L_-arginine methyl ester (L-NAME). NOS activity was calculated as the amount of _L_-[^3^H]-arginine converted to _L_-[^3^H]-citrulline (in disintegrations.min^-1^).min^-1^.mg protein^-1^.

### Statistical Analysis

Relative body mass changes over the 14 day statin supplementation period was analyzed using a two-way repeated measures ANOVA, and when a significant interaction was observed, with Bonferoni post-hoc test. Three group comparisons between control and the two statin groups were analyzed using a one-way ANOVA with Newman-Keul’s post-hoc test. Statistical significance was set at p < 0.05. All data are presented as mean ± SEM. The statistical software package GraphPad Prism 5.00 was utilized for all statistical analysis.

## Results

### Plasma Creatine Kinase and Total Cholesterol

**Fig 1:**
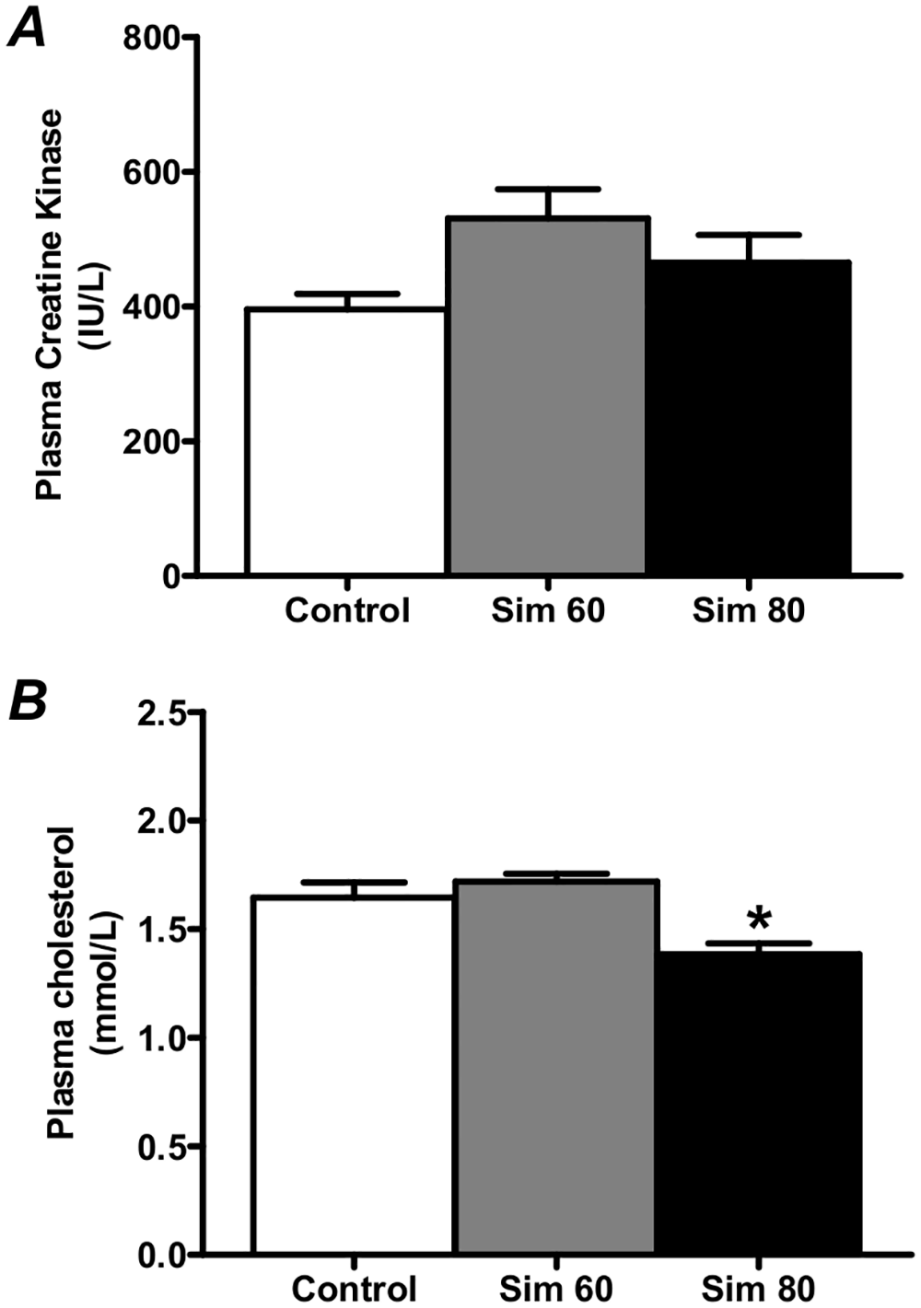
The simvastatin on plasma creatine kinase (CK) activity and total cholesterol. Rats were treated with vehicle (Control) or simvastatin at 60 (Sim 60) and 80 mg.kg^-1^.day^-1^ (Sim 80) for 14 days. (**A**) Plasma CK activity. (**B**) Plasma [total cholesterol]. *—significantly different from Control and Sim 60 groups. Mean ± SEM. n = 8/group. One way ANOVA with Newman-Keul’s post-hoc test. P < 0.05.

Previous studies have shown that simvastatin at 88 mg·kg^-1^·day^-1^, but not at 80 mg·kg^-1^·day^-1^, induces significant damage in rat skeletal muscle that is accompanied by very large increases in plasma CK activity (over 300-fold increase), an indirect marker of muscle damage [16]. Therefore, in order to examine the effect of simvastatin, independent of overt muscle damage and regeneration, we chose simvastatin doses [60 mg.kg^-1^·day^-1^ (Sim 60) and 80 mg·kg^-1^·day^-1^ (Sim 80). Our results confirm that 14 d of simvastatin treatment induced only very minor increases in plasma CK activity (1.34- and 1.18-fold for the Sim 60 and Sim 80 groups, respectively; p = 0.054, main effect, Fig 1A) compared to Control animals. Furthermore, plasma [total cholesterol] was only reduced in the Sim 80 group compared to the Control (Fig 1B). These data show that, although simvastatin (80 mg.kg^-1^.day^-1^) significantly decreased plasma cholesterol, in agreement with the study of Mallinson *et al*. (2009), this dose induced only very minor increases in plasma CK activity. Moreover, these results suggest that our simvastatin treatments did not elicit overt skeletal muscle damage and regeneration.

### Body Mass

**Fig 2:**
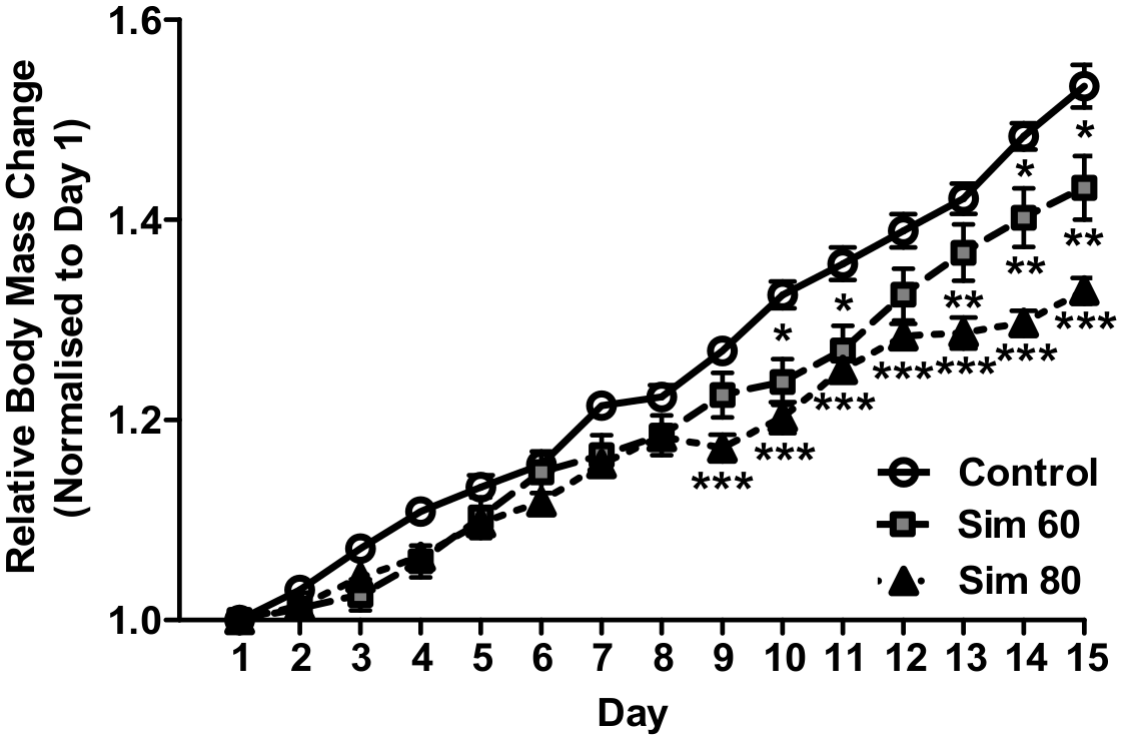
The effect of simvastatin treatment on body mass. Rats were treated with vehicle (Control) or simvastatin at 60 (Sim 60) and 80 mg.kg^-1^.day^-1^ (Sim 80) for 14 days. Body mass was normalized to each rat’s initial starting mass (day 0). *—Sim 60 significantly different from Control. **—Sim 80 significantly different from Sim 60. ***—Sim 80 significantly different from Control. Mean ± SEM. n = 8/group. Two way ANOVA with repeated measures and Bonferonni’s post-hoc test. P < 0.05.

Throughout the 2 wk treatment period, rats administered simvastatin exhibited no visible signs of lethargy, altered gait or ruffled fur that would indicate marked statin toxicity or reduced mobility. Furthermore, there were no significant differences in food consumption between the groups (S2 Fig). There were, however, significant main effects for the relative increase in body mass for simvastatin treatment and time, coupled with a significant interaction effect (Fig 2). As such, by the end of treatment, the increase in body mass of the Sim 60 and Sim 80 groups was 10% and 20% lower than the controls, respectively, with the increase in Sim 80 being lower than Sim 60 over the last 6 days of treatment.

### Muscle Atrophy-Related Gene Expression

**Fig 3:**
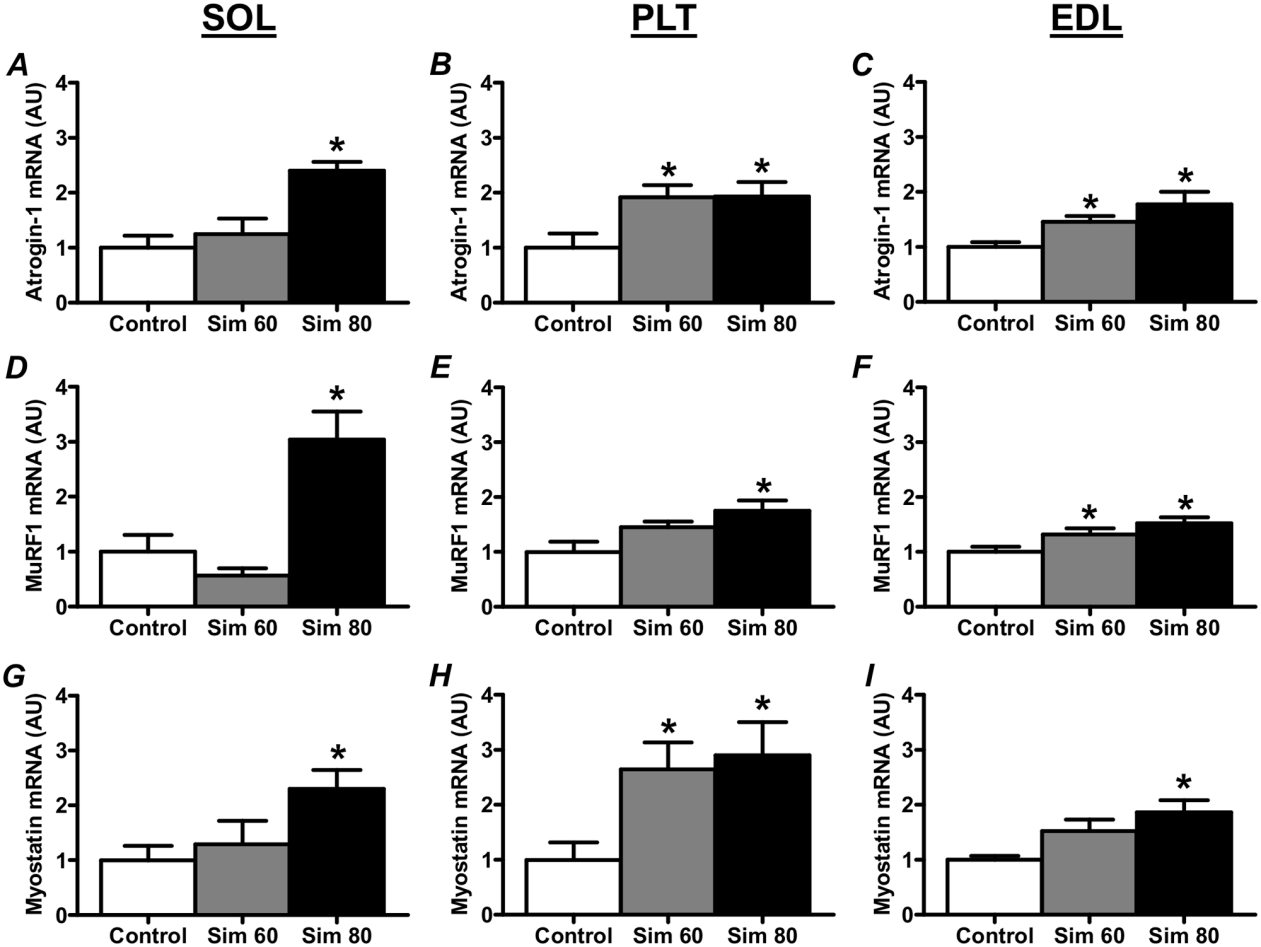
The effect of simvastatin treatment on the expression of muscle atrophy-related genes, Atrogin-1, MuRF1 and Myostatin. Rats were treated with vehicle (Control) or simvastatin at 60 (Sim 60) and 80 mg.kg^-1^.day^-1^ (Sim 80) for 14 days. Muscle mRNA is extracted and analyzed as described in the Methods. mRNA expression was normalised to total cDNA. *—significantly different from Control. Mean ± SEM. n = 6–8/group. One way ANOVA with Newman-Keul’s post-hoc test. P < 0.05.

As shown in Fig 3, there was a significant increase in the expression of atrogin-1 and MuRF1 mRNA in the Sim 80 groups of all three muscles (Fig 3A–3F). Furthermore, there were significant increases in atrogin-1 mRNA in the PLT and EDL Sim 60 groups (Fig 3B and 3C) and in MuRF1 mRNA in the EDL Sim 60 group (Fig 3F).

A potential upstream regulator of atrogin-1 and MuRF1 expression is the TGF super family member, myostatin [55,56]. Previous studies have shown that statins can increase the expression of another TGF super family member, TGF-β, and increase TGF-β signaling, in bone and smooth muscle cells [57,58]. Thus, we sought to determine whether simvastatin would also induce an increase in the expression of myostatin in skeletal muscle. Indeed, myostatin mRNA was increased in the Sim 80 groups of all three muscles and also in the PLT Sim 60 group (Fig 3G–3I).

### PGC-1α, NRF-1 and Tfam Protein Expression

**Fig 4:**
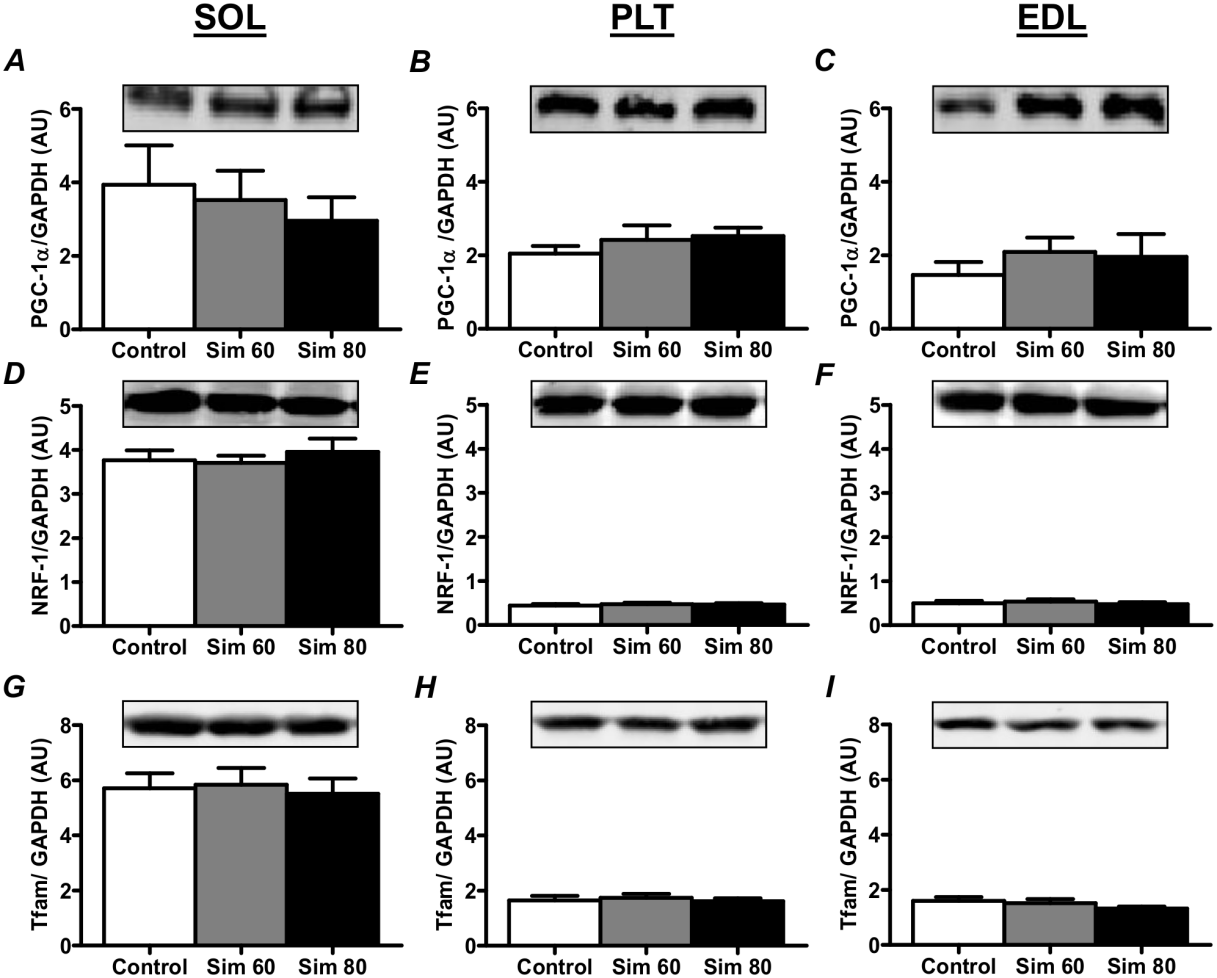
The effect of simvastatin treatment on the PGC1α, NRF-1 and Tfam protein expression. Rats were treated with vehicle (Control) or simvastatin at 60 (Sim 60) and 80 mg.kg^-1^.day^-1^ (Sim 80) for 14 days. Muscles were collected and subjected to Western blot analysis with the indicated antibodies as described in the Methods. Values were normalised to GAPDH and expressed as arbitrary units (AU). Mean ± SEM. n = 8/group. One way ANOVA with Newman-Keul’s post-hoc test.

Simvastatin had no effect on PGC1α protein in any of the muscle types (Fig 4A–4C). This is further supported by the finding that there was no change in the protein expression of two downstream PGC1α targets, NRF-1 (Fig 4D–4F) and Tfam (Fig 4G–4I).

### Markers of Mitochondrial Volume

**Fig 5:**
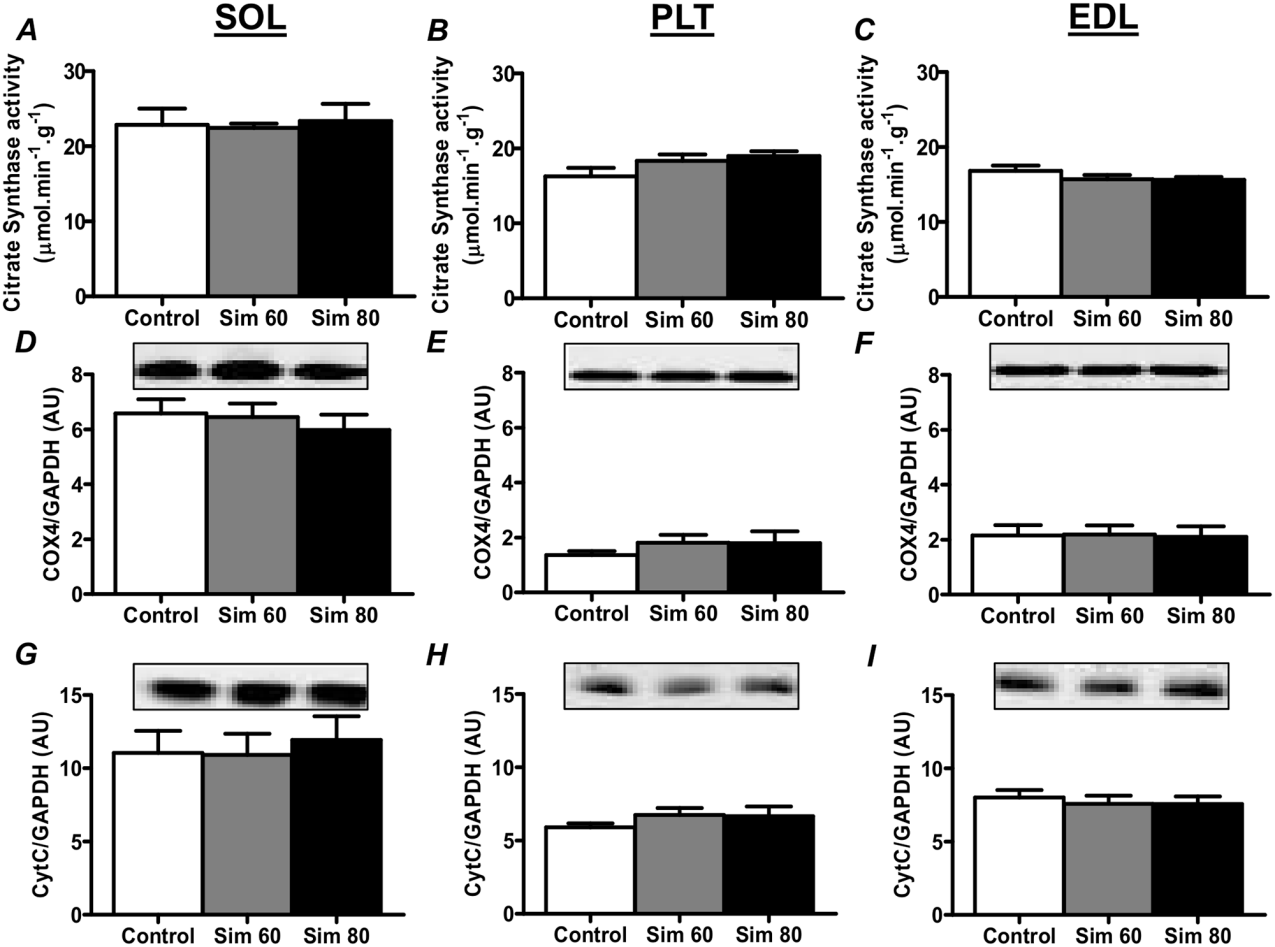
The effect of simvastatin treatment on citrate synthase activity, and Cox4 and Cyt C protein expression. Rats were treated with vehicle (Control) or simvastatin at 60 (Sim 60) and 80 mg.kg^-1^.day^-1^ (Sim 80) for 14 days. Muscles were collected and subjected to enzyme activity analysis or Western blot analysis with the indicated antibodies as described in the Methods. Protein expression values were normalised to GAPDH and expressed as arbitrary units (AU). Mean ± SEM. n = 8/group. One way ANOVA with Newman-Keul’s post-hoc test.

The activity of citrate synthase (CS), a mitochondrial matrix enzyme from the Kreb’s Cycle, is a commonly used marker of oxidative capacity/mitochondrial number [e.g. [59]]. Therefore, we examined the effect of simvastatin treatment on CS activity and found no change in CS activity in all three muscles (Fig 5A–5C). Furthermore, we found no changes in the expression of electron transport chain proteins, cytochrome c oxidase subunit 4 (Cox4; Fig 5D–5F) and cytochrome c (CytC; Fig 5H–5J).

### Beta-Hydroxyacyl-CoA-Dehydrogenase (-HAD) Activity

**Fig 6:**
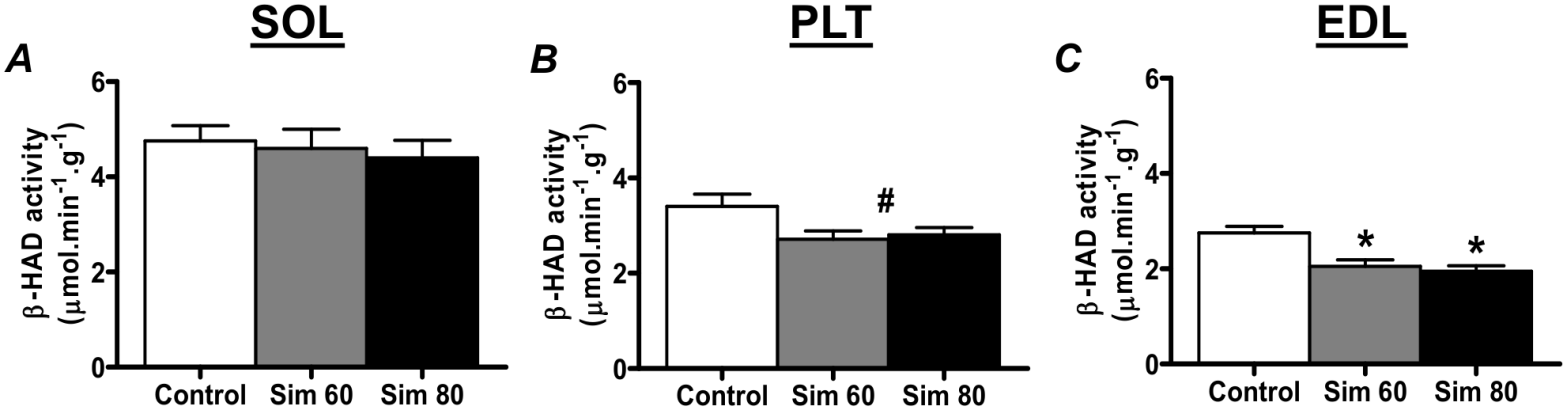
The effect of simvastatin treatment on β-HAD activity. Rats were treated with vehicle (Control) or simvastatin at 60 (Sim 60) and 80 mg.kg^-1^.day^-1^ (Sim 80) for 14 days. Muscles were collected and subjected to enzyme activity analysis as described in the Methods. *—significantly different from Control (P < 0.05). #—strong trend for main effect (P = 0.05) compared to Control. Mean ± SEM. n = 8/group. One way ANOVA with Newman-Keul’s post-hoc test.

We next determined the effect of simvastatin on the activity of beta-hydroxyacyl-CoA-dehydrogenase (-HAD), an enzyme of the fatty acid -oxidation pathway. As shown in Fig 6, simvastatin induced a significant decrease in -HAD activity in the EDL Sim 60 and Sim 80 groups (Fig 6C), with a strong trend (p = 0.05, main effect) for a decrease in the PLT Sim 60 and Sim 80 groups (Fig 6B). There was no effect of simvastatin on -HAD activity in the SOL muscle.

### AMPK

**Fig 7:**
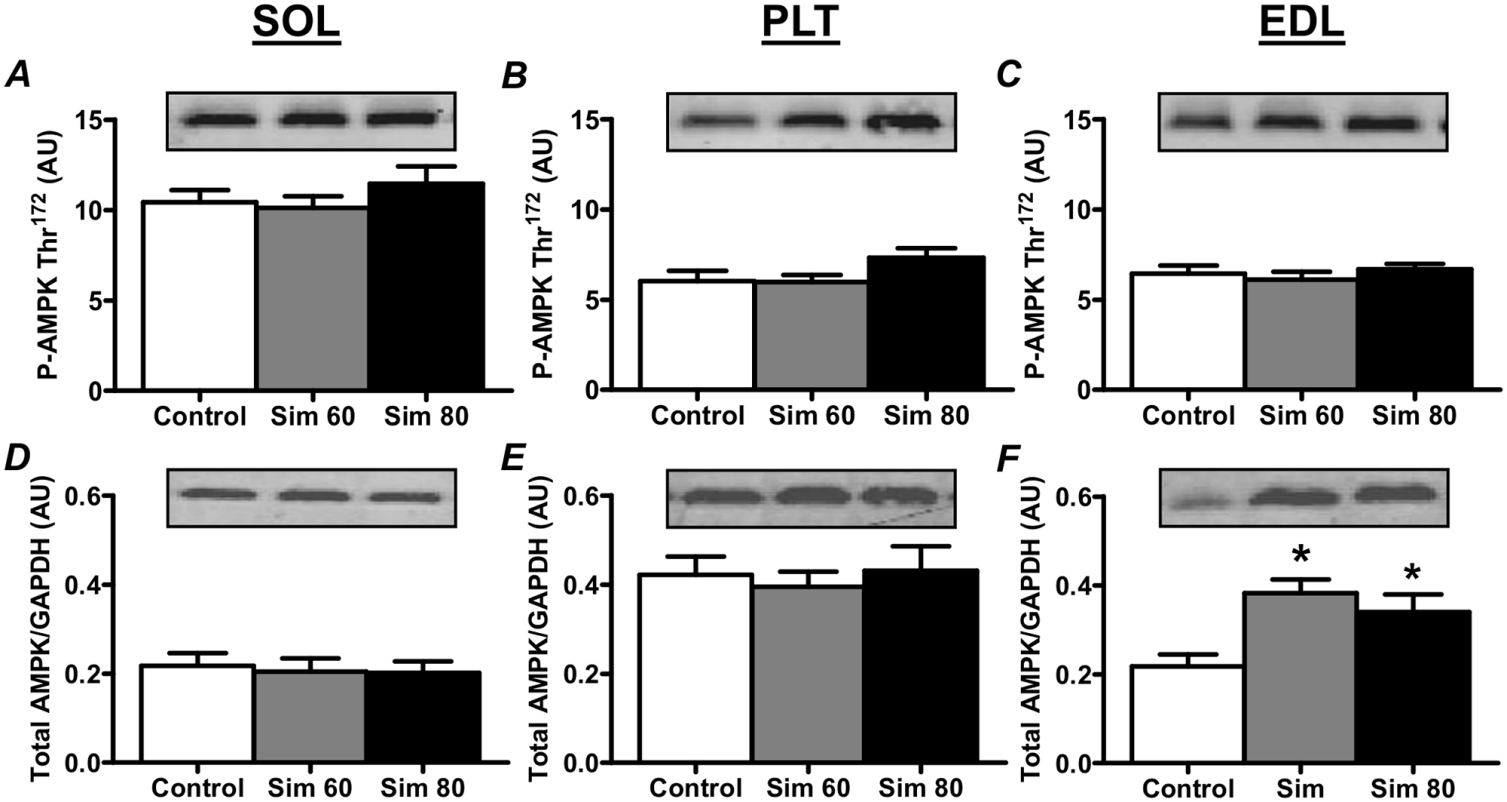
The effect of simvastatin treatment on the AMPK Thr^172^ phosphorylation and total AMPK protein expression. Rats were treated with vehicle (Control) or simvastatin at 60 (Sim 60) and 80 mg.kg^-1^.day^-1^ (Sim 80) for 14 days. Muscles were collected and subjected to Western blot analysis with the indicated antibodies as described in the Methods. Values for total AMPK were normalised to GAPDH. All values are expressed as arbitrary units (AU). *—significantly different from Control. Mean ± SEM. n = 8/group. One way ANOVA with Newman-Keul’s post-hoc test. P < 0.05.

There was no difference in AMPKα Thr^172^ phosphorylation compared to Controls in all three muscles (Fig 7A–7C). Simvastatin treatment did, however, induce an increase in total AMPKα protein expression in the Sim 60 and Sim 80 groups of the EDL muscle (Fig 7F) but not in the SOL or PLT muscles (Fig 7D and 7E).

### Nitric Oxide Synthase

**Fig 8:**
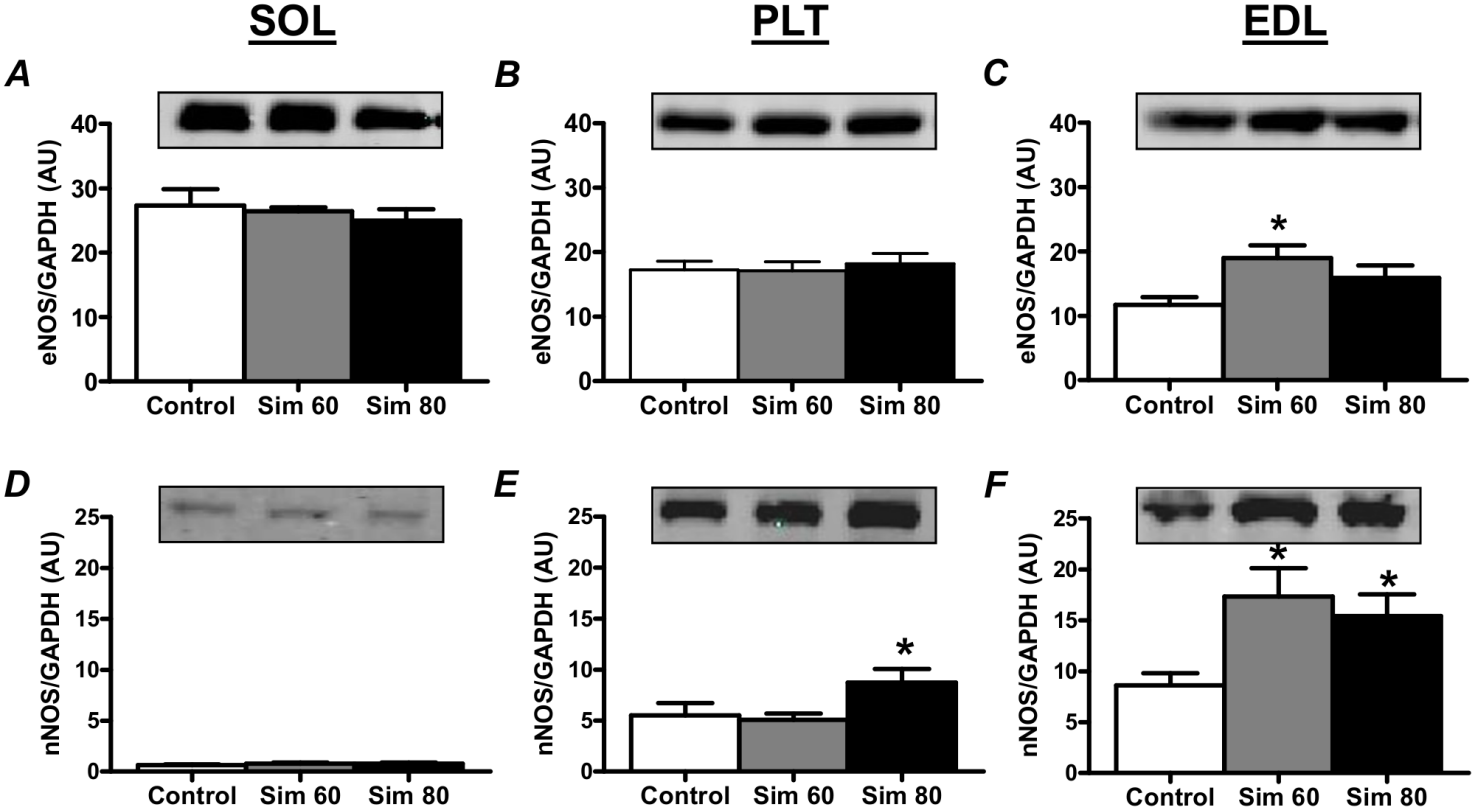
The effect of simvastatin treatment on the nNOS and eNOS protein expression. Rats were treated with vehicle (Control) or simvastatin at 60 (Sim 60) and 80 mg.kg^-1^.day^-1^ (Sim 80) for 14 days. Muscles were collected and subjected to Western blot analysis with the indicated antibodies as described in the Methods. Values were normalised to GAPDH and expressed as arbitrary units (AU). *—significantly different from Control. Mean ± SEM. n = 6–8/group. One way ANOVA with Newman-Keul’s post-hoc test. P < 0.05.

Simvastatin induced an increase in eNOS protein expression in the EDL Sim 60 group (Fig 8C) and increased nNOS protein expression in the EDL Sim 60 (Fig 8F) and PLT and EDL Sim 80 groups (Fig 8E and 8F). Given that simvastatin induced increases in eNOS and nNOS occurred predominantly in the EDL muscle we next wanted to determine whether there would also be a corresponding increase in total NOS activity in this muscle. Indeed, while not statistically significant, total NOS activity in the EDL groups closely mirrored the changes in NOS protein expression (see S3 Fig).

## Discussion

In this study, we sought to examine the relationship between the statin-induced increase in the expression of the muscle atrophy-related genes, mitochondrial biogenesis-related signaling molecules and markers of mitochondrial content. Our results show for the first time that simvastatin increases the expression of UPS E3-ligase genes, atrogin-1 and MuRF1, not only in predominantly fast-twitch/glycolytic muscles, but also in predominantly slow-twitch/oxidative muscle. Furthermore, our results demonstrate that simvastatin also induces an increase in the expression of TGF-β superfamily member, myostatin, in both fast- and slow-twitch muscles. Importantly, we show that the expression of these atrophy-related genes was not associated with changes in PGC1α protein, or with changes in markers of mitochondrial volume in either muscle type. Finally, we show for the first time that simvastatin induced an increase in total AMPK, eNOS and nNOS protein expression, and decreased the activity of fatty acid β-oxidation enzyme, β-HAD in fast-twitch but not in slow-twitch muscle.

PGC1α is a major regulator of mitochondrial biogenesis and a recent study has reported that patients with statin myopathy have reduced muscle PGC1α mRNA expression [23]. Furthermore, 2 wk of statin treatment has been shown to reduce PGC1α mRNA expression in rat skeletal muscle, while reductions in PGC1α mRNA and protein have been reported in cultured cells [23,32]. A statin-induced decrease in PGC1α could help to explain reports that statin myopathy is associated with a reduction in mitochondrial content [14,23,30–32,60] and an increase in the activation of atrophy genes and muscle atrophy [14,34,35]. Our findings, however, show that 2 wk of simvastatin treatment did not alter PGC1α protein expression (or the expression of downstream targets, NRF-1 and Tfam) or markers of mitochondrial content (e.g. Cox4 and Cyt C protein expression and CS activity), despite an increase in the expression of atrogin-1, MuRF1 and myostatin. These findings show that the statin-induced increase in atrophy gene expression occurs prior to any changes in PGC1α protein expression and mitochondrial content and suggest that other factors play a more important role in the initial statin-induced activation of atrophy genes.

One factor such that could play a role in the activation of atrophy gene expression is statin-induced mitochondrial dysfunction [17–29]. Indeed, Mallinson et al. recently reported that the statin-induced increase in atrogin-1 mRNA was associated with an up regulation of the known FoxO1 gene target pyruvate dehydrogenase kinase-4 (PDK4) expression, decreased pyruvate dehydrogenase complex (PDC) activity and increased muscle glycogen content [16,18]. Importantly, pharmacological activation of the PDC by chronic dichloroacetate (DCA) treatment was sufficient to blunt the up regulation of atrogin-1 mRNA [18]. Combined, these studies suggest that statins may induce a decrease in glucose oxidation which could ultimately lead to glucose intolerance, insulin resistance and the activation of FoxO target genes that include atrophy-related genes [61–63]. An increase in muscle lipid content may also play a role in the etiology of skeletal muscle insulin resistance [64]. As such, it is interest to note we show for the first time that *in vivo* statin treatment reduced -HAD activity in fast-twitch muscle. Previous studies have also reported that simvastatin increases LDL receptor content, LDL uptake, and lipoprotein lipase (LPL) activity in skeletal muscle [65,66]. Thus, a statin-induced increase in LDL uptake and LPL activity, combined with a statin-induced reduction in the capacity for -oxidation of fatty acids, could also lead to a toxic lipid overload which may play a role in the eventual development of insulin resistance and the activation of muscle atrophy genes, especially in fast-twitch muscle. Additional studies are therefore needed to further investigate the molecular interaction between statins and mitochondrial fatty acid metabolism *in vivo*, and the relationship with muscle atrophy gene expression.

AMPK is an important activator of mitochondrial biogenesis [67] and a potential activator of myostatin, atrogin-1 and MuRF1 expression [44,68]. Numerous studies have shown that statins induce the activation of AMPK *in vivo* and in cultured cells (e.g. [36–39]), including in L6 muscle cells [69]. In the present study, however, we found no evidence of sustained AMPK activation 24 h after the last statin treatment, as indicated by the absence of an increase in AMPK Thr^172^ phosphorylation. This result could mean that there was no statin-induced activation of AMPK or that any statin-induced activation of AMPK was transient and had returned to baseline by 24 h. This later possibility is supported by the study of Sun *et al*. (2008) that showed that the *in vivo* statin-induced increase in aortic and cardiac tissue AMPK activity peak at ~4 h and had largely returned to baseline by 24 h. It remains unknown, however, whether repeated transient activations of AMPK would be sufficient to induce skeletal muscle atrophy gene expression *in vivo*. Clearly, if such activation did occur, it was not sufficient to alter mitochondrial content. In this study, we also found that total AMPK protein was increased by statin treatment in fast-twitch muscle. The implication of this finding is unclear but it may suggest the potential for a greater AMPK signaling in response to a given stimulus. Further research is therefore required to clarify the short and long term effects, if any, of statins on skeletal muscle AMPK activity and muscle metabolism.

Studies in non-muscle cells have also established that statins promote an increase in eNOS mRNA, protein content and activity, and NO production in endothelial tissue [70,71], in part, via the inhibition of RhoA geranylgeranylation [for review see [72]]. This statin-induced inhibition of RhoA geranylgeranylation leads to inhibition of RhoA membrane translocation and activity, a decrease in Rho-associated kinase (ROCK) activity, and an increase in eNOS mRNA stability that results in elevated levels of eNOS protein [40,73]. More recently, statins have also been shown to increase nNOS mRNA and protein expression in non-skeletal muscle tissues/cells, and this was also associated with reduced ROCK activity [42,43,74]. In the current study, we demonstrate for the first time that statins can also induce increases in both eNOS and nNOS protein in fast-twitch skeletal muscle. This result provides strong indirect evidence that our simvastatin treatment induced an inhibition of RhoA/ROCK activity in fast-twitch muscle. This finding is also consistent with a previous study that detected an early simvastatin-induced inhibition of RhoA activity in rat fast-twitch muscle [16]. The reason for the lack of change in eNOS/nNOS expression in the SOL muscle is, however, unclear. Perhaps, given that the basal expression of nNOS (the major NOS isoform expressed in skeletal muscle cells) is very low in slow-twitch muscles (see Fig 8D), other more dominant regulatory mechanisms may operate in rat slow-twitch muscle to limit the up regulation of nNOS protein. Whatever the mechanism, further research is warranted to explore the metabolic implications of the statin-induced increase in NOS/NO in fast-twitch muscles.

## Conclusion

In conclusion, our results show that in the absence of overt muscle damage, statin-induced increases in muscle atrophy gene expression occurred independently of changes in the protein expression of PGC1α, or with changes in markers of mitochondrial content. This study also demonstrated for the first time that simvastatin treatment was sufficient to induce an increase in AMPK, eNOS and nNOS protein expression, and to decrease the activity of fatty acid β-oxidation enzyme, β-HAD, in fast- but not slow-twitch muscle. These fast-twitch muscle specific changes may represent early events that could, in part, contribute to the previously reported development of overt muscle damage in fast-twitch muscles/fibers.

## Supporting Information

S1 Fig Representative western blot images of GAPDH protein expression in Control and Simvastatin treated muscles. Rats were treated with vehicle (C) or simvastatin at 60 (S60) and 80 (S80) mg.kg^-1^.day^-1^ for 14 days. Muscles were collected and subjected Western blot analysis as described in the Methods.
(TIF)

S2 Fig Mean daily (S2 Fig 2A), and total (S2 Fig 2B), food consumption by Control and Simvastatin treated rats. Rats were treated with vehicle (Control) or simvastatin at 60 (Sim 60) and 80 (Sim 80) mg.kg^-1^.day^-1^ for 14 days. Rats were housed in groups in 4 animals per cage. Food was weighed prior to each daily dose of vehicle or simvastatin for 14 days. Mean ± SEM. A. Two way ANOVA with Bonferoni’s post-hoc test (P > 0.05). B. One way ANOVA with Newman-Keul’s post-hoc test (P > 0.05).
(TIF)

S3 Fig The effect of simvastatin treatment on the EDL total NOS activity. Rats were treated with vehicle (Control) or simvastatin at 60 (Sim 60) and 80 mg.kg^-1^.day^-1^ (Sim 80) for 14 days. EDL muscles were collected and subjected to nitric oxide synthase (NOS) activity analysis as described in the Methods. Mean ± SEM. n = 4–7/group. There was no significant differences between any of the groups. One way ANOVA with Newman-Keul’s post-hoc test. P = 0.231.
(TIF)
