# Supplementary figures and images for: Statin-Induced Increases in Atrophy Gene Expression Occur Independently of Changes in PGC1α Protein and Mitochondrial Content
Source: PLoS One. 2015 May 28;10(5):e0128398. doi: 10.1371/journal.pone.0128398 (PMC4447258; doi:10.1371/journal.pone.0128398)

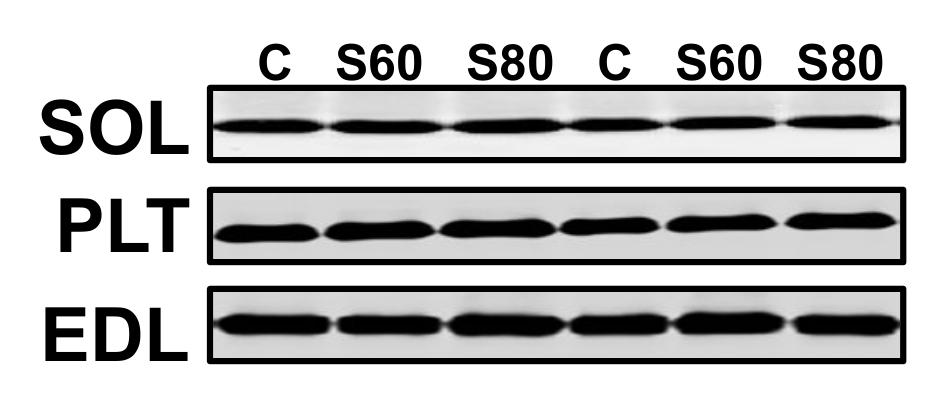

Supplement: S1 Fig — Rats were treated with vehicle (C) or simvastatin at 60 (S60) and 80 (S80) mg.kg-1.day-1 for 14 days. Muscles were collected and subjected Western blot analysis as described in the Methods. (TIF) [file pone.0128398.s001.tif]

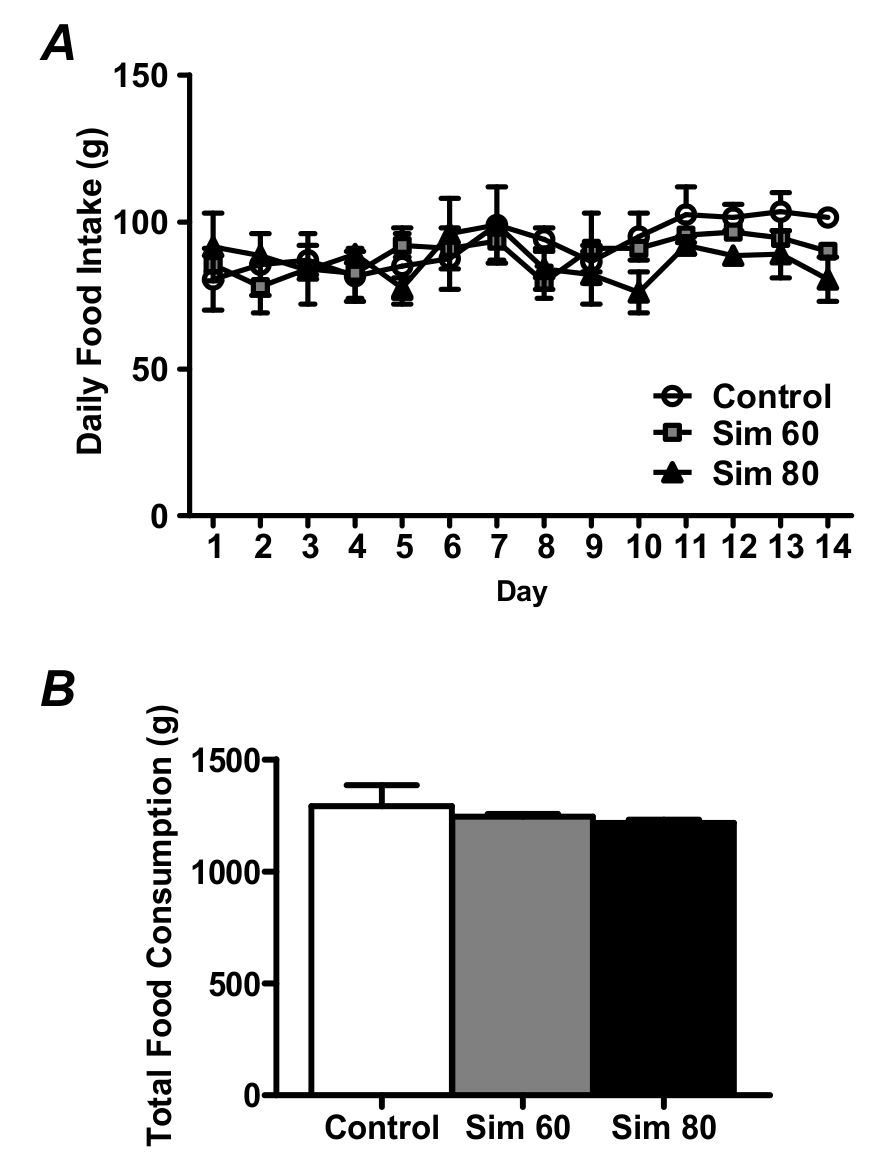

Supplement: S2 Fig — Rats were treated with vehicle (Control) or simvastatin at 60 (Sim 60) and 80 (Sim 80) mg.kg-1.day-1 for 14 days. Rats were housed in groups in 4 animals per cage. Food was weighed prior to each daily dose of vehicle or simvastatin for 14 days. Mean ± SEM. A. Two way ANOVA with Bonferoni’s post-hoc test (P > 0.05). B. One way ANOVA with Newman-Keul’s post-hoc test (P > 0.05). (TIF) [file pone.0128398.s002.tif]

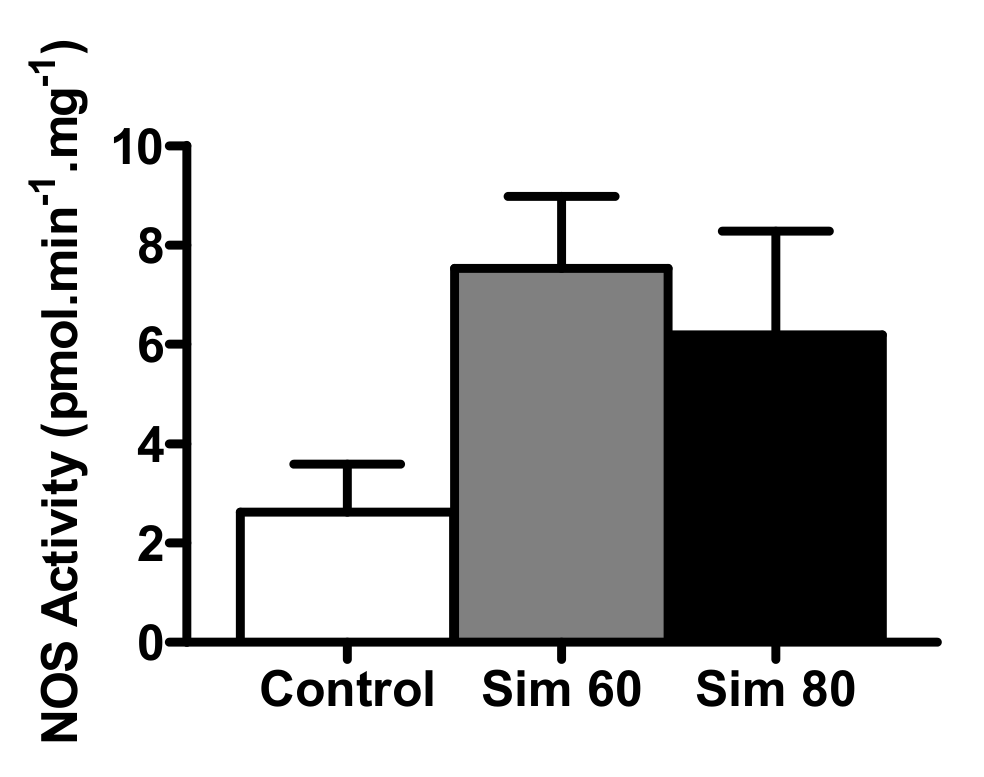

Supplement: S3 Fig — Rats were treated with vehicle (Control) or simvastatin at 60 (Sim 60) and 80 mg.kg-1.day-1 (Sim 80) for 14 days. EDL muscles were collected and subjected to nitric oxide synthase (NOS) activity analysis as described in the Methods. Mean ± SEM. n = 4–7/group. There was no significant differences between any of the groups. One way ANOVA with Newman-Keul’s post-hoc test. P = 0.231. (TIF) [file pone.0128398.s003.tif]
